# Fibromyalgia in men: beyond invisibility—a biopsychosocial and clinical perspective in rheumatology

**DOI:** 10.1007/s10067-026-08184-x

**Published:** 2026-05-25

**Authors:** André Pontes-Silva

**Affiliations:** Postgraduate Program in Physical Education, Federal University of Maranhão, São Luís, MA Brazil

**Keywords:** Biological differences, Female disease, Fibromyalgia

## Abstract

Fibromyalgia is a chronic pain syndrome traditionally perceived as a predominantly female condition, although growing evidence suggests that this view may partly reflect diagnostic and sociocultural biases rather than true epidemiological differences. This perspective critically examines fibromyalgia in men as an underrecognized and understudied clinical entity. The authors propose an integrative conceptual framework in which the invisibility of men with fibromyalgia emerges from the interaction between sociocultural barriers to healthcare-seeking, clinician-related diagnostic bias, and heterogeneous symptom expression influenced by biological and psychosocial factors. Men may delay seeking medical care due to cultural expectations of masculinity and frequently encounter skepticism regarding symptoms such as diffuse pain, fatigue, and psychological distress. Although core symptoms are shared across sexes, men may differ in coping strategies, illness perception, and healthcare interaction. Emerging evidence also suggests possible neurophysiological and functional alterations, including small fiber pathology, reduced muscular strength, gait impairments, and psychological comorbidities. However, current evidence remains limited by small samples and methodological heterogeneity. The article argues that understanding fibromyalgia in men is essential not to establish rigid sex-specific phenotypes, but to improve diagnostic sensitivity, promote biopsychosocial and gender-sensitive care, and expand the understanding of heterogeneity within chronic pain disorders.

Fibromyalgia is a chronic pain syndrome characterized by widespread musculoskeletal pain, fatigue, sleep disturbance, cognitive impairment, and a variety of somatic and psychological symptoms [1]. Despite decades of research, fibromyalgia remains a controversial and frequently misunderstood condition within clinical medicine [2]. One of the most persistent assumptions surrounding the syndrome is that it is primarily a disorder affecting women. Indeed, clinical samples and epidemiological reports consistently show a predominance of female patients [3]. However, this perception may reflect diagnostic practices and research bias rather than true epidemiology [4].

## Aim of the perspective

The aim of this perspective is to critically examine fibromyalgia in men as an underrepresented clinical entity and to propose an integrated conceptual framework that incorporates epidemiological, sociocultural, biological, and clinical dimensions. By doing so, we seek to move beyond descriptive synthesis and provide a structured interpretation with direct clinical and research implications. To achieve this objective, we first examine epidemiological and sociocultural factors, followed by biological and clinical dimensions, and finally discuss implications for practice and research.

**Fig. 1:**
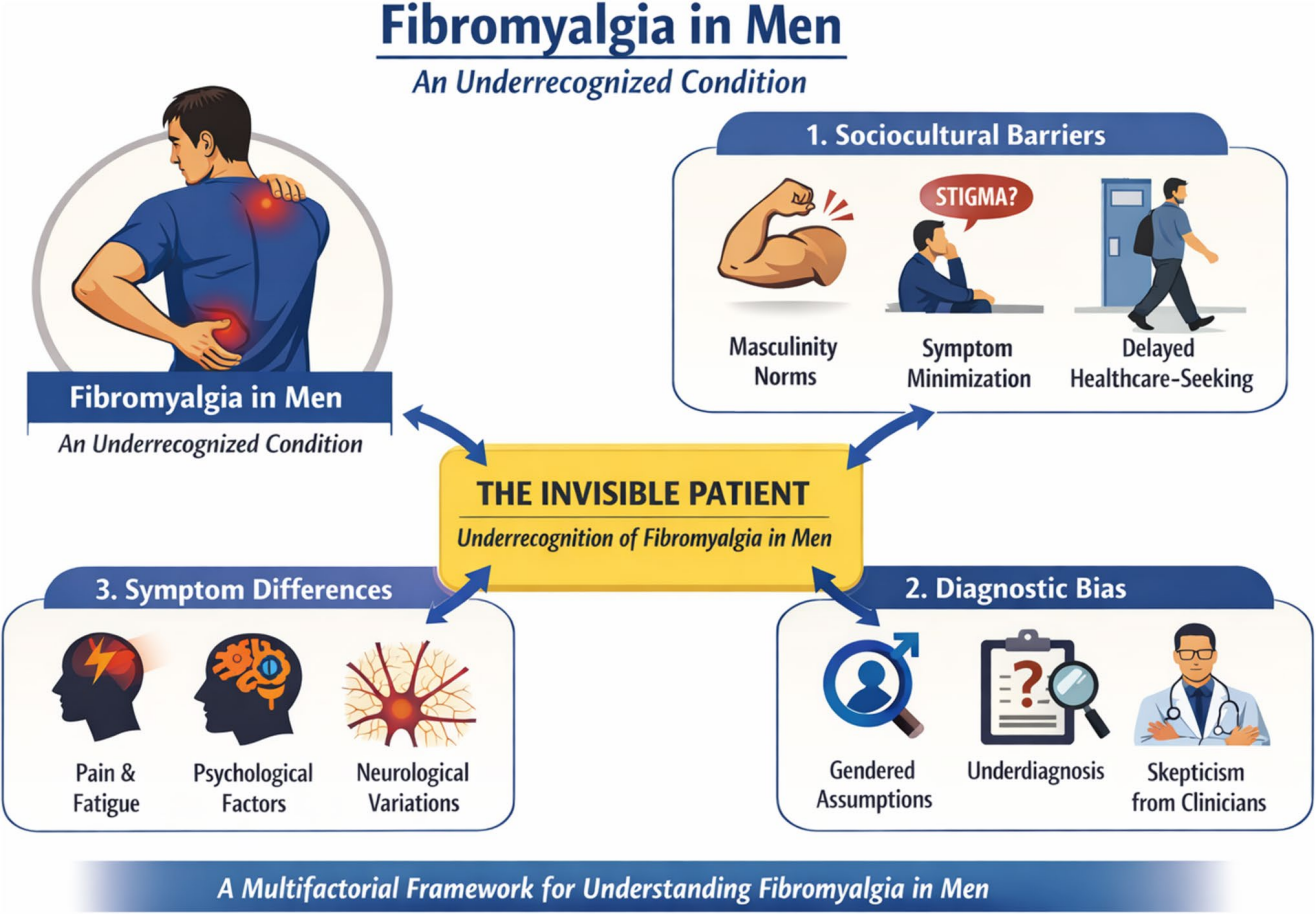
Conceptual framework of fibromyalgia in men: interactions between sociocultural factors, diagnostic bias, and symptom expression

In this perspective, we argue that fibromyalgia in men represents a systematically invisible clinical population, resulting from the interaction between diagnostic bias, sociocultural norms of masculinity, and incomplete biomedical models of chronic pain. This invisibility not only contributes to delayed diagnosis and suboptimal care but also constrains the scientific understanding of fibromyalgia as a heterogeneous condition [5]. Building on this argument, we propose a conceptual framework in which fibromyalgia in men emerges from the dynamic interaction between (i) sociocultural barriers to healthcare-seeking, (ii) clinician-related diagnostic bias, and (iii) sex-related differences in symptom expression, ultimately leading to underrecognition and underrepresentation in research (Fig. 1).

## Epidemiology and diagnostic bias

Historically, fibromyalgia has been framed as a “female disease,” with women accounting for the vast majority of diagnosed cases. Early clinical cohorts reported that between 80 and 90% of patients were women, reinforcing this perception within both the medical community and the general public. Nevertheless, closer examination of population-based studies suggests that the gender gap may not be as extreme as previously believed. Some estimates indicate that men may represent a substantially larger proportion of individuals with fibromyalgia than clinical data suggest. The discrepancy between population prevalence and clinical diagnosis may indicate a process of systematic underrecognition in men rather than true rarity [6]. However, these estimates remain heterogeneous and should be interpreted cautiously.

## Sociocultural determinants and healthcare-seeking behavior

The underdiagnosis of fibromyalgia in men may also be influenced by sociocultural factors related to gender norms. Men with chronic pain conditions often delay seeking medical care, particularly when symptoms involve fatigue, diffuse pain, or psychological distress. Such symptoms may conflict with cultural expectations of masculinity, which emphasize physical strength and resilience. Consequently, many men may attempt to manage their symptoms independently rather than pursue medical evaluation. Qualitative research examining the lived experiences of male patients suggests that men frequently encounter skepticism from healthcare professionals and social networks when describing their symptoms. This skepticism may further discourage healthcare-seeking behavior and reinforce the invisibility of fibromyalgia among men [6]. These findings, although derived from limited qualitative data, point to potential barriers in healthcare access and validation.

## Clinical phenotype and sex-based comparisons

Beyond diagnostic disparities, emerging research suggests that fibromyalgia in men may present with distinct clinical characteristics. Although core symptoms such as widespread pain and fatigue appear similar between sexes, several studies have identified differences in psychological profiles, comorbid conditions, and coping mechanisms. For example, men with fibromyalgia may experience different patterns of pain-coping strategies and interpersonal behaviors compared with female patients [5]. However, women with fibromyalgia have been more extensively studied and are also known to present high levels of pain, fatigue, psychological distress, and functional impairment, indicating substantial overlap between sexes.

Current evidence therefore supports the existence of both shared core features and potential sex-related variations, rather than clearly distinct phenotypes. A more nuanced interpretation may therefore be that fibromyalgia expresses a shared core pathophysiology across sexes, while sociocultural and biological factors modulate symptom interpretation, communication, coping strategies, and clinical presentation. In this sense, sex-related differences may emerge less as distinct disease entities and more as variations in illness experience and healthcare interaction.

This interpretation aligns directly with the conceptual framework proposed in this perspective, in which sociocultural influences, diagnostic practices, and heterogeneous symptom expression interact dynamically to shape the clinical visibility of fibromyalgia in men. Rather than supporting a rigid biological dichotomy between sexes, the available evidence suggests that differences may emerge through distinct pathways of symptom communication, healthcare access, illness interpretation, and clinical recognition.

## Biological mechanisms

Biological differences may also contribute to sex-specific manifestations of fibromyalgia. Recent neurophysiological investigations have begun to explore peripheral nervous system involvement in male patients. In particular, studies assessing small fiber pathology have identified abnormalities in intraepidermal nerve fiber density and sensory processing among men diagnosed with fibromyalgia [7]. However, it is important to note that small fiber pathology has also been consistently reported in women with fibromyalgia, and therefore does not represent a sex-specific mechanism. Rather, these findings support the broader concept that fibromyalgia may involve heterogeneous biological mechanisms across patients, irrespective of sex. In male cohorts, reduced corneal nerve fiber density, altered thermal detection thresholds, and impairment of C-tactile afferents have been observed, indicating potential involvement of small nerve fibers in symptom generation. These findings suggest, but do not confirm, that neuropathic mechanisms may contribute to symptom generation in a subset of patients.

Importantly, the current evidence supporting small fiber involvement in men with fibromyalgia remains methodologically limited. Most available studies include relatively small samples, cross-sectional designs, and lack appropriate sex-balanced comparison groups, which restricts causal inference and generalizability. Furthermore, abnormalities in small fiber structure or function are not specific to fibromyalgia and have also been described in other chronic pain conditions, raising questions regarding their diagnostic specificity. Thus, while these findings may support the hypothesis that peripheral nociceptive alterations contribute to symptom heterogeneity, they should not yet be interpreted as definitive biomarkers of male fibromyalgia. Future studies integrating neurophysiological, histopathological, and longitudinal clinical assessments are needed to clarify whether these alterations represent disease mechanisms, epiphenomena, or adaptive responses to chronic pain. Standardization of diagnostic criteria, sex-balanced sampling strategies, and longitudinal follow-up protocols will also be essential to improve comparability and external validity across future studies [8].

Another important and underexplored dimension relates to the role of sex hormones in fibromyalgia. Although this perspective focuses on men, current evidence suggests that hormonal factors—including testosterone, estrogen, and their interactions with pain modulation systems—may contribute to sex-related differences in symptom perception and central sensitization. These hormonal influences have been more extensively studied in women, particularly in relation to estrogen fluctuations, whereas their role in men remains insufficiently characterized.

In addition, current evidence on hormonal influences in fibromyalgia is marked by substantial heterogeneity. Variations in testosterone levels, hypothalamic–pituitary–adrenal axis activity, and neuroendocrine stress responses have been inconsistently reported across studies. Many investigations are limited by inadequate control of confounding variables such as age, obesity, sleep quality, physical activity, medication use, and psychiatric comorbidities, all of which may independently affect endocrine function. Consequently, although hormonal mechanisms remain biologically plausible, their specific contribution to fibromyalgia in men remains uncertain and should be interpreted cautiously [9].

Importantly, the search for sex-related biological distinctions in fibromyalgia should avoid reductionist interpretations that attribute clinical differences exclusively to biological sex. Pain perception, symptom reporting, stress responses, and healthcare-seeking behaviors are also shaped by sociocultural and psychological processes, which may interact bidirectionally with neurobiological mechanisms. Therefore, current evidence may be more consistent with a multidimensional model of sex- and gender-influenced variability rather than with clearly separated male and female pathophysiological phenotypes [10].

## Functional and physical impairments

In addition to neurophysiological alterations, functional impairments have also been documented in men with fibromyalgia. Objective measures of physical capacity indicate that male patients often exhibit reduced muscular strength compared with healthy controls. For instance, studies assessing handgrip strength have reported lower values among men with fibromyalgia [11]. Although reduced handgrip strength is also well documented in women with fibromyalgia, its clinical relevance in men may deserve particular attention because muscular performance and physical capacity are often central components of masculine identity and occupational functioning.

Consequently, reductions in strength may produce distinct psychosocial and functional consequences in men, potentially influencing healthcare-seeking behavior, illness perception, and disability experience. From a clinical perspective, objective functional measures such as handgrip strength may therefore provide useful complementary information in the assessment of male patients with suspected fibromyalgia. Therefore, the potential utility of handgrip strength should be interpreted as a general functional marker in fibromyalgia, not restricted to male patients [11].

Motor function and biomechanical alterations represent another underexplored aspect of fibromyalgia. Investigations into gait dynamics have demonstrated that male patients may exhibit slower walking speed, reduced stride length, and lower cadence compared with healthy individuals [12]. Although gait impairments are not exclusive to men, investigating locomotor function in male patients may still be clinically informative. Functional limitations involving walking performance, cadence, and mobility may be particularly relevant in men due to occupational demands, physical role expectations, and patterns of symptom interpretation. Therefore, the importance of gait analysis may reside less in identifying sex-specific abnormalities and more in understanding how fibromyalgia-related functional impairment manifests within different social and behavioral contexts. These observations are consistent across small studies but require confirmation in larger and more diverse samples.

## Psychological and sociocultural dimensions

Psychological comorbidities also appear to play a significant role in the clinical presentation of fibromyalgia in men. Anxiety, depression, and other affective disorders are common among patients with chronic pain conditions [13]. While these conditions are highly prevalent in both men and women with fibromyalgia, differences may exist in how symptoms are expressed, reported, and socially perceived. Recent research has also explored the role of identity and self-perception in shaping the psychological experiences of men with fibromyalgia [14]. Given that this area is still emerging, these interpretations should be considered exploratory.

## Treatment and therapeutic considerations

The impact of fibromyalgia on daily functioning and quality of life in men is substantial [15]. Many patients report dissatisfaction with pharmacological treatments and limited symptom relief [16]. Lifestyle interventions have shown promising results [17]. However, current evidence on treatment response remains largely derived from predominantly female samples, limiting the ability to draw firm conclusions regarding sex-specific effectiveness. It is plausible that men may differ in treatment adherence, response patterns, or tolerability, particularly in interventions involving psychological or behavioral components, but this hypothesis requires further investigation.

Clinically, treatment approaches for men with fibromyalgia may benefit from greater emphasis on functional goals, physical performance, and shared decision-making strategies. Some male patients may demonstrate lower acceptance of interventions perceived as predominantly psychological, which reinforces the importance of integrating education, exercise-based rehabilitation, and symptom validation within interdisciplinary care models. Tailoring communication strategies to improve engagement without reinforcing gender stereotypes may represent an important component of patient-centered care [18, 19].

Taken together, these findings suggest that fibromyalgia in men represents a complex and underrecognized clinical phenomenon. Early investigations demonstrated that men may experience significant symptoms and functional limitations [20], while more recent studies have identified potential biological and psychosocial distinctions [21]. However, the current body of evidence remains limited and fragmented.

## Conceptual and clinical implications

Based on the available evidence, we propose a clinically oriented framework in which fibromyalgia in men should be understood as a multidimensional condition shaped by three interacting domains: (i) delayed healthcare-seeking driven by sociocultural norms, (ii) diagnostic bias within clinical settings, and (iii) heterogeneous symptom expression influenced by biological and psychosocial factors. This framework suggests that underdiagnosis is not merely a patient-related issue but a systemic phenomenon involving both patients and healthcare systems.

Importantly, this framework does not require the assumption that fibromyalgia in men constitutes a biologically distinct subtype. Rather, it proposes that differences in recognition, symptom expression, and healthcare trajectories may emerge from the interaction between shared pain mechanisms and gender-influenced psychosocial processes. This distinction is clinically relevant because it shifts the focus from searching for exclusively male biomarkers toward improving diagnostic sensitivity and person-centered assessment strategies.

Accordingly, the originality of the present framework lies in proposing that the invisibility of fibromyalgia in men should not be understood merely as a consequence of insufficient biological evidence, but rather as the product of interacting sociocultural, clinical, and research-related processes that collectively shape disease recognition. This integrative interpretation may help bridge traditionally separated biological and psychosocial models of fibromyalgia.

For clinicians, particularly rheumatologists, this perspective has several implications. Screening strategies should be broadened to actively consider fibromyalgia in men presenting with non-specific chronic pain. Clinicians should be aware that men may underreport symptoms or express them differently. Structured assessment tools and objective functional measures may improve diagnostic accuracy. In rheumatology practice, clinicians should consider incorporating routine screening for diffuse pain, fatigue, sleep disturbances, and psychosocial distress in male patients presenting with persistent musculoskeletal complaints, even when inflammatory or structural findings are absent. Greater attention to illness narratives, functional limitations, and symptom duration may help reduce diagnostic delays and unnecessary investigations.

For example, male patients presenting repeatedly with persistent diffuse musculoskeletal pain, fatigue, non-restorative sleep, and inconclusive imaging or laboratory findings may benefit from earlier fibromyalgia-oriented assessment rather than repeated investigation focused exclusively on structural pathology. Similarly, incorporating simple functional measures, such as handgrip strength, walking performance, or patient-reported fatigue scales, may help clinicians identify clinically relevant impairment beyond traditional rheumatologic examination findings. Furthermore, explicitly validating patients’ pain experiences may be particularly important in men, who may minimize symptoms or avoid discussing emotional distress due to sociocultural expectations related to masculinity [22].

A biopsychosocial approach that explicitly addresses gender-related barriers may improve patient engagement and treatment adherence. From a physiotherapy perspective, individualized rehabilitation programs emphasizing gradual progression of physical activity, pacing strategies, pain education, and functional restoration may be particularly relevant for male patients with fibromyalgia. Considering occupational demands, exercise expectations, and potential resistance to psychologically oriented interventions may improve therapeutic alliance and long-term adherence. In addition, integrating patient education regarding central sensitization and chronic pain mechanisms may help reduce stigma and facilitate self-management behaviors [23].

## Future directions

Future research should move beyond descriptive comparisons and focus on three priority areas. First, large-scale epidemiological studies are needed to better estimate the true prevalence of fibromyalgia in men and to clarify the extent of diagnostic bias. Second, mechanistic studies should investigate whether biological differences, including small fiber pathology and central pain processing, differ meaningfully between sexes. Third, clinical trials should examine whether men respond differently to pharmacological and non-pharmacological interventions, and whether tailored treatment strategies are warranted.

Additionally, qualitative and mixed-method research should continue to explore the role of masculinity, identity, and stigma in shaping illness experiences. Integrating these perspectives into clinical and experimental research may contribute to a more comprehensive understanding of fibromyalgia as a gender-influenced condition.

## Literature selection

This perspective is based on a targeted, non-systematic review of the literature. Studies were identified through searches in databases such as PubMed and Scopus, focusing on fibromyalgia in men, sex differences, and related biopsychosocial factors. Searches were conducted using combinations of terms including “fibromyalgia,” “men,” “male patients,” “sex differences,” “gender,” “chronic pain,” “small fiber pathology,” “psychosocial factors,” and “rehabilitation.” Preference was given to peer-reviewed studies published in English and indexed in major biomedical databases. Reference lists of relevant articles were also manually screened to identify additional studies pertinent to the scope of this perspective.

Particular attention was given to studies discussing male-specific experiences, sex-related clinical comparisons, chronic pain mechanisms, healthcare access, rehabilitation, and biopsychosocial dimensions of fibromyalgia. When conflicting findings were identified, preference was given to interpretations supported by broader conceptual consistency and methodological transparency rather than isolated statistically significant results.

Priority was given to recent publications and studies including male-specific analyses. Due to the limited availability of male-focused research, evidence from mixed-sex samples was also considered when relevant (Table 1). The literature selection process was guided primarily by conceptual relevance to the proposed framework rather than by formal systematic review methodology. Accordingly, studies were included when they contributed to understanding epidemiological, sociocultural, biological, functional, or clinical dimensions of fibromyalgia in men. Given the limited availability of male-focused investigations, the inclusion of exploratory and mixed-sex studies was considered necessary to contextualize current knowledge gaps and emerging hypotheses.

**Table 1:** Summary of key studies on fibromyalgia in men: clinical, biological, and psychosocial findings

Study (year)	Design/sample	Population	Main findings in men	Key contribution	Limitations
Drusko et al. (2023) [Clinical Phenomenology of Fibromyalgia Syndrome in Male Patients: Same But Different]	Observational	Men with fibromyalgia	Differences in coping strategies and interpersonal behavior compared to women	Suggests psychosocial distinctions in male patients	Small sample; cross-sectional
Ruschak et al. (2023) [Fibromyalgia Syndrome Pain in Men and Women: A Scoping Review]	Scoping review	Mixed (men and women)	Evidence of underrecognition and gender-related diagnostic bias	Highlights sociocultural and diagnostic factors	Heterogeneous studies
Feulner et al. (2024) [Pain and small fiber pathology in men with fibromyalgia syndrome]	Experimental/neurophysiology	Men with fibromyalgia	Altered small fiber function and sensory processing	Suggests neuropathic component in subset of patients	Limited sample size
Aparicio et al. (2010) [Handgrip strength in men with fibromyalgia]	Cross-sectional	Men with fibromyalgia	Reduced handgrip strength vs controls	Indicates functional impairment	Older study; small sample
Heredia-Jimenez et al. (2014) [Kinematics gait disorder in men with fibromyalgia]	Biomechanical analysis	Men with fibromyalgia	Slower gait, reduced stride length and cadence	Demonstrates motor dysfunction	Small sample; limited generalizability
Henao-Pérez et al. (2022) [Patients With Fibromyalgia, Depression, and/or Anxiety and Sex Differences]	Observational	Mixed	High psychological burden; possible sex differences	Supports role of mental health	Inconsistent sex comparisons
Geller & Levy (2026) [Masculine Identity, Body Image and Illness-Related Shame: Pathways to Psychological Distress in Men with Fibromyalgia]	Observational	Men with fibromyalgia	Illness challenges masculine identity; linked to distress	Introduces identity-based framework	Early-stage evidence
Ruschak et al. (2022) [Symptomatology of Fibromyalgia Syndrome in Men: A Mixed-Method Pilot Study]	Mixed-method	Men with fibromyalgia	Severe symptoms, dissatisfaction with treatment	Provides patient-centered insights	Pilot study; small sample
Kueny et al. (2021) [Fibromyalgia Pain and Fatigue Symptoms in Spanish and U.S. Men]	Cross-cultural	Men (Spain vs USA)	Cultural context influences symptom perception	Highlights sociocultural variability	Limited generalizability
Salar et al. (2023) [Client-centered lifestyle intervention for men with fibromyalgia syndrome: Is efficacy independent of gender?]	Intervention study	Men and women	Lifestyle interventions improve outcomes	Suggests treatment potential	Not male-specific
Buskila et al. (2000) [Fibromyalgia syndrome in men]	Observational	Men with fibromyalgia	Men experience significant symptoms and disability	Early evidence of male involvement	Older study
Bannon et al. (2025) [Regarding the pain of men: characteristics of fibromyalgia in male patients]	Observational	Men with fibromyalgia	Confirms clinical burden and characteristics in men	Reinforces emerging evidence	Limited sample

## Limitations

The available evidence on fibromyalgia in men is limited by small sample sizes, cross-sectional designs, and underrepresentation of male participants in most cohorts. Additionally, heterogeneity in diagnostic criteria and study methodologies complicates comparisons across studies. These limitations restrict the strength of inferences and highlight the need for more robust, longitudinal, and male-focused research. Additionally, the non-systematic nature of this perspective limits reproducibility and may introduce selection bias in the interpretation of the literature. Although the narrative approach was intentionally chosen to support conceptual integration and critical reflection, it does not provide the methodological rigor or comprehensiveness of systematic reviews or meta-analyses. Consequently, the proposed framework should be interpreted as a hypothesis-generating and clinically oriented synthesis rather than definitive evidence regarding sex-specific mechanisms or treatment responses in fibromyalgia. As a result, current interpretations regarding sex differences should be considered provisional and hypothesis-generating rather than definitive.

The persistent invisibility of fibromyalgia in men has important implications for both clinical practice and research. This invisibility should be understood as a systemic phenomenon rather than an isolated diagnostic issue, arising from the interaction between sociocultural norms, healthcare practices, and research priorities. Diagnostic bias may delay appropriate treatment, while limited representation in clinical studies restricts understanding of disease mechanisms and therapeutic responses.

Current evidence does not support the existence of a completely distinct male phenotype of fibromyalgia. Nevertheless, the persistent underrepresentation of men in clinical cohorts and experimental studies suggests that important dimensions of symptom experience, healthcare access, and treatment response remain insufficiently understood. Thus, the relevance of studying fibromyalgia in men lies not in establishing rigid sex dichotomies, but in expanding the understanding of heterogeneity within chronic pain conditions. Recognizing fibromyalgia in men is not merely a matter of gender equity; it is essential for advancing the broader understanding of chronic pain disorders. A shift toward integrative, biopsychosocial, and gender-sensitive models of research and care may help address current gaps and improve outcomes for this underrecognized population.

## Data Availability

N/A.
